# Knowledge and Practices Related to Salt Intake among Saudi Adults

**DOI:** 10.3390/ijerph17165749

**Published:** 2020-08-09

**Authors:** Mahitab A. Hanbazaza, Walaa A. Mumena

**Affiliations:** 1 Department of Food and Nutrition, Faculty of Human Sciences and Design, King Abdulaziz University, P.O. Box 80200, Jeddah 21589, Saudi Arabia; mhanbazaza@kau.edu.sa; 2 Clinical Nutrition Department, College of Applied Medical Sciences, Taibah University, P.O. Box 344, Madinah 42353, Saudi Arabia

**Keywords:** knowledge, practices, salt intake, adults, Saudi Arabia

## Abstract

In Saudi Arabia, data regarding salt-related knowledge and practices are still lacking. This cross-sectional study aimed to investigate salt-related knowledge and practices and associated factors in Saudi adults. Data on the following variables were collected from 467 participants living in Madinah or Jeddah via face-to-face interviews: demographics, anthropometrics (height and weight), blood pressure (assessed using a digital sphygmomanometer), salt-related knowledge, and practices related to salt intake. Salt-related knowledge and practices were limited among the study participants; however, they were not correlated (r_s_ = 0.10). Multiple linear regression analysis revealed that salt-related practices were negatively associated with sodium intake and positively associated with body mass index (BMI) (*p* < 0.001 and *p* = 0.001, respectively), whereas salt-related knowledge was not associated with sodium intake, blood pressure, or BMI. Salt-related knowledge is limited and not linked to practices related to salt intake in Saudi adults. Interventions are needed to increase the accessibility of low-sodium food options and improve practices limiting sodium intake to prevent the occurrence of salt-related diseases among adults in Saudi Arabia.

## 1. Introduction 

Salt is a common form of sodium, and previous studies have revealed that it is consumed excessively in many populations, including that of Saudi Arabia [1,2]. High levels of sodium consumption are associated with many chronic diseases, such as hypertension (HTN) and cardiovascular disease (CVD) [3,4,5]. In fact, excessive sodium intake is known as a silent killer, and it is responsible for approximately 2.5 million deaths annually [6,7]. In Saudi Arabia, CVD is one of the most common causes of death [8]. To limit sodium intake and prevent salt-related diseases, several organizations recommend sodium intake limits. For example, the American Heart Association (AHA) recommends restraining sodium intake to less than 2300 mg of sodium per day [9].

Food sources of sodium vary across populations [5]. Despite the differences in the eating habits of individuals, processed and restaurant-prepared food have been reported to largely contribute to high sodium intake [10,11]. In the Gulf Cooperation Council countries (Saudi Arabia, Bahrain, Kuwait, the United Arab Emirates, Oman, and Qatar), bread was determined to be the principal source of salt in the diets of individuals in 2015 [12]. Furthermore, recent evidence has shown that a large proportion of the food consumed by Saudis is processed food that is high in sodium [2,13].

Other factors such as sex and awareness of the health consequences of excessive intake of sodium are also linked to excessive sodium intake [14]. Limited awareness of the health consequences of excessive sodium intake has been reported in a number of populations [2,15]. Salt-related knowledge is important, because it likely affects the practices associated with reducing the levels of salt in the diet [16,17]. Reducing salt intake significantly decreases the risk of CVD and stroke, improves health outcomes, and reduces mortality rates [18,19]. Improving an individual’s understanding of healthy eating habits and the health consequences of a poor diet has been found to be an effective means of influencing eating behaviors and practices [20]. 

The World Health Organization (WHO) recommends the evaluation of individuals’ knowledge and practices related to salt intake in order to plan interventions that aim to reduce salt intake [20]. In Saudi Arabia, data regarding salt-related knowledge and practices are still lacking. Thus, this study aimed to investigate salt-related knowledge and practices and the associated factors among Saudi adults.

## 2. Materials and Methods 

### 2.1. Sample

This cross-sectional study included 494 healthy participants aged between 20 and 50 years who were recruited conveniently between January and March 2020 from two large cities, Madinah and Jeddah, in the Western region of Saudi Arabia. Participants were recruited from public places in both cities, including malls, parks, Jeddah Corniche, and walking paths. Male participants from Madinah who expressed interest in participating in the study, but were not able to provide data at the time, were invited to visit the Home Care Service Center in Madinah, Saudi Arabia. Pregnant women; individuals with diabetes, HTN, or CVD; and individuals with a history of bariatric surgery, those following a special diet, or those taking any medication that might influence blood pressure or weight were excluded. Individuals who reported participating in moderate- or high-intensity physical activity at the time of data collection were also excluded. Data on all participants were collected via face-to-face interviews at data collection stations. Participants were asked to provide data regarding their age, sex (male; female), marital status (single; married), education level (high-school/diploma; university degree/postgraduate degree), family income per month (<SR 5000; SR 5000–10,000; >SR 10,000), and city of residence (Jeddah; Madinah). Ethical approval for the study was obtained from the Ethical Committee of the College of Applied Medical Sciences, Taibah University (certificate number: SREC/AMS 2019/33/CND). Signed consent forms were obtained from all participants prior to data collection.

### 2.2. Sodium Intake

Dietary sodium intake was assessed using a semi-validated food frequency questionnaire (FFQ) [21]. The FFQ was used to evaluate the quantity of sodium naturally present in food and the quantity added during cooking, but not sodium coming from table salt. The FFQ was specifically developed to assess sodium intake in the Saudi population. The sodium content in food was estimated by the Nutritics software^®^ (version 5.09, Libro, Dublin, Ireland). Information of local food items and recipes were manually added into the Nutritics software. The sodium contents of all listed food items were validated based on the sodium contents of local products.

### 2.3. Assessment of Anthropometric Measurements 

The height and weight of each participant were measured thrice using standardized procedures, and the average values were recorded. Weight was assessed using a portable electronic scale (Omron, BF508, Japan), and values were recorded to the nearest 0.1 kg. Height was assessed using a measuring tape placed on a straight wall, and values were recorded to the nearest 0.5 cm. Weight and height measurements were used to estimate the body mass index (BMI) of each participant, and weight status was determined in accordance with the WHO criteria [22]. All participants were provided with cards containing the following information: date of data collection, height, weight, BMI, and blood pressure readings. 

### 2.4. Assessment of Blood Pressure 

A digital sphygmomanometer (Cardio Simple, Pic Solutions, Casnate Con Bernate, Italy) was used to assess blood pressure after it was calibrated and validated appropriately. Blood pressure was measured using standardized procedures after participants had been instructed to relax and sit calmly for 5 minutes. Blood pressure measurements were taken thrice at 5-minute intervals, and the average readings were recorded. Blood pressure cutoff values were specified based on 17 American College of Cardiology (ACC)/AHA guidelines: normal blood pressure are <120/80 mm Hg; elevated blood pressure, systolic blood pressure values between 120 and 129 mm Hg and diastolic blood pressure values <80 mm Hg; stage 1 hypertension, systolic blood pressure values between 130 and 139 mm Hg or diastolic blood pressure values between 80 and 89 mm Hg; stage 2 hypertension, systolic blood pressure values of at least 140 mm Hg or diastolic blood pressure values of at least 90 mm Hg [23]. If high blood pressure was observed, participants were advised to visit a physician to perform additional tests, receive counsel, and receive treatment if needed.

### 2.5. Assessment of Salt-Related Knowledge 

Salt-related knowledge was assessed using the following 5 questions that were adapted from a study conducted by Ismail et al. (2019) (accounting for a total score of 5) [24]: 1) Could high salt intake adversely affect health? 2) Could high salt intake increase the risk of HTN? 3) Could high salt intake increase the risk of osteoporosis? 4) Could high salt intake increase the risk of stomach cancer? and 5) Could high salt intake increase the risk of kidney stones? Participants were allowed to select between “yes” or “no” when answering each question. Correct answers to all questions were “yes”. Variables were converted to numerical scale where a score of one was assigned to the total knowledge score variable for every correct answer. When participants gave a wrong answer, a score of zero was assigned. 

### 2.6. Assessment of Salt-Related Practices

Practices related to salt intake were assessed based on positive behaviors with respect to 6 items that were also adapted from Ismail et al. (accounting for a total score of 6) [24]. Item one focused on sodium intake; a score of one was assigned if the participant’s sodium intake was within the limit recommended by the AHA (<2300 mg/day), whereas a score of zero was assigned the participant’s sodium intake that exceeded the sodium recommendation. The second item focused on the addition of salt during cooking; a score of one was assigned if salt was never, rarely, or sometime added to the food during cooking, while a score of zero was assigned if salt was frequently added to the food during cooking. The third item focused on the use of table salt; a score of one was assigned if salt was never, rarely, or sometime added at the table, while a score of zero was assigned if salt was frequently added at the table. Responses to items two and three were “never”; “rarely”; “sometime”; “usually”; and “always”. The fourth item focused on the effort made to reduce salt intake; a score of one was assigned if the participant made an effort to reduce salt intake, while a score of zero was assigned if the participant did not report making an effort to reduce salt intake. The fifth item focused on the use of salt alternatives; a score of one was assigned if the participant reported using salt alternatives, while a score of zero was assigned if the participant did not report using salt alternatives. Responses to items 4 and 5 were “yes” and “no”. The sixth item focused on limiting fast food consumption; a score of one was assigned if the frequency of fast food consumption was ≤1 instance/week, while a score of zero was assigned if the frequency of fast food consumption was ≥2 instance/week. To assess the frequency of fast food consumption, participants were asked to report the frequency of their usual fast food consumption in a typical week.

### 2.7. Statistical Analysis 

Descriptive data were presented as means (standard deviations (SD)) for symmetric continuous variables, medians (interquartile ranges (IQR)) for asymmetric continuous variables, and frequencies (percentages (%)) for categorical variables. A chi-square test was used to assess whether there was an association between two categorical variables. Spearman correlation was used to assess the associations between the total scores of salt-related knowledge and salt reduction practices. The and Kruskal–Wallis and Mann–Whitney U tests were used to assess whether the differences across groups were significant. Multiple linear regression analysis was performed to determine the association between the total score of salt-related knowledge and sodium intake, blood pressure, and BMI after adjusting for sex (male = 1 and female = 2). In addition, multiple linear regression models were used to explore the association between the total score of salt-related practices and sodium intake, blood pressure, and BMI. These models were adjusted for age, sex (male = 1 and female = 2), marital status (single = 0 and married = 1), and city of residence (Madinah = 1 and Jeddah = 2). The assumptions of multiple linear regression analysis were evaluated and met. All tests performed were two-tailed and a significance level of 0.05 was used. Statistical analyses were performed using the Statistical Package for the Social Sciences (SPSS 20, SPSS Inc, Chicago, IL, USA). 

## 3. Results 

The total number of healthy participants included in this study was 467 after the exclusion of participants with missing data (n = 27, 5.47%). Over three-quarters of the participants (n = 368) were recruited from Madinah. The mean age of the study participants was 29.0 (9.10) years, and 60% (n = 281) were female. The median sodium intake was 4273 (IQR: 3053–5979) mg/day. The mean BMI was 25.86 (5.81) kg/m^2^, which was categorized as overweight. The mean systolic blood pressure and diastolic blood pressure values were 119 (12.0) and 80.6 (9.32) mm Hg, respectively. A detailed description of the sociodemographic and physical characteristics of the study participants is provided in Table 1. 

The median salt-related knowledge score was 3.00 (IQR 3.00–4.00) out of 5.00, whereas the median salt-related practices score was 1.00 (IQR 1.00–2.00) out of 6.00. Spearman correlation between salt-related knowledge and practices was weak (r_s_ = 0.10, *p* = 0.036). An assessment of salt-related knowledge and practices according to sex revealed that higher proportion of female participants recognized the effect of high salt intake on health (*p* < 0.001). In addition, higher proportion of female participants responded correctly to the questions regarding high salt intake and the increased risk of HTN and kidney stones (*p* = 0.006 and *p* < 0.001, respectively). Lower proportion of female participants reported frequent consumption of fast food (*p* < 0.001), as shown in Table 2.

Only 11 participants (2.36%) scored 5 out of 5 on the assessment of salt-related knowledge; 168 (36.0%) participants scored 4 out of 5. One participant (0.21%) scored 5 out of 5 on the assessment of items concerning practices related to salt intake, and 13 participants (2.78%) scored 4 out of 5. The scores of salt-related knowledge and practices were significantly higher among female participants than among male participants (*p* < 0.001). The scores of practices related to salt intake were significantly higher among older participants (age ≥40 years) and among participants who were obese (*p* < 0.001 and *p* = 0.001, respectively). Additionally, participants who were married and those living in Madinah had high mean scores for salt-related practices (*p* < 0.001 and *p* = 0.043, respectively). The associations between salt-related knowledge and practices and the characteristics of participants are presented in Table 3. 

Multiple linear regression analysis revealed that salt-related practice scores were negatively associated with sodium intake and positively associated with BMI, whereas salt-related knowledge scores were not associated with sodium intake, blood pressure, or BMI (Table 4). 

## 4. Discussion

Limited salt-related knowledge and practices were observed among the study sample. Salt-related knowledge was not associated with sodium intake, blood pressure, or BMI, whereas practices related to salt intake were negatively associated with sodium intake and positively associated with BMI.

Our findings suggest that single individuals could have better practices related to salt intake, compared with married individuals. This differed from the findings of a previous study, in which no association was reported between marital status and increased sodium consumption [25]. In addition, females in our study reported better salt-related knowledge and practices than males. Similar findings were reported in a review conducted in 2018, in which females were found to have enhanced salt-related knowledge. The study also showed that females made greater effort to reduce sodium levels by reducing salt intake and buying less fast food [26]. In fact, women were found to be more health conscious overall and were more likely to read and follow nutritional recommendations than men [27,28]. 

Findings of this study also suggest that older participants may have better salt-related practices than younger participants. These data are in accordance with the findings of previous studies, which have also shown that older people have better dietary practices [15,27,28,29]. Older individuals tend to be more health conscious, and that may be because health-related topics are less important to young individuals. As individuals age, their health declines and health-related topics become increasingly important [30]. 

Obesity or higher BMI of individuals included in this study was associated with better practices related to salt. In a study conducted in China, individuals who realized that they were at an increased risk of health consequences due to excessive sodium intake were more likely to prioritize the reduction of sodium intake [29]. Correspondingly, it has been reported that people who are aware of their current positive health status tend not to worry about salt intake [31]. 

No association between salt-related knowledge and sodium intake, blood pressure, or BMI was found in this study, which might reflect changes occurring in the Saudi diet. The Saudi diet has been influenced by urbanization, which has caused a shift from a traditional diet to a more westernized diet that is high in salt, added sugar, fat, and calories [32,33]. In addition, this lack of associations may indicate that the majority of individuals in the Saudi population have increased access to high-salt foods, such as processed or restaurant-prepared foods [32]. In fact, over 63% (n = 295) of the participants included in this study consumed fast food more than twice a week. It is also possible that participants were unaware of the upper limits specified for sodium intake or failed to recognize that particular foods contain high levels of sodium, such as fast food [15].

A high level of awareness regarding diseases linked to excessive salt intake has been reported previously; however, despite this level of awareness, the intake of high levels of sodium persists [15,29]. This study reported limited salt-related knowledge as well as poor practices toward reducing salt intake. In addition, no correlation was found between salt-related knowledge and practices. This clearly indicates that salt-related knowledge may not be the only factor influencing practices related to salt intake. The association between salt-related knowledge and practices is complicated and may be influenced by several factors, including demographics, educational level, economic status, the environment, and culture [34,35,36].

To our knowledge, this is the first study to assess salt-related knowledge and practices among Saudi adults. However, the findings of this study might not be generalizable to the overall population of Saudi Arabia, as we performed convenience sampling. In this study, sodium intake was assessed by the FFQ. Despite the fact that the estimate of sodium intake that is obtained via a 24-h urine sample is considered the gold standard, the FFQ can provide a good estimate of the sodium intake of a group [21]. In addition, data concerning awareness regarding the food sources of sodium and the ability to read nutrition labels were not collected in this study. 

## 5. Conclusions

Salt-related knowledge is limited and not linked to practices related to salt intake among Saudi adults. Our findings highlight the need of establishing interventions that aim to limit salt consumption by increasing the accessibility of low-sodium food options and improving practices related to salt intake. In fact, increasing public awareness regarding salt intake, using innovative approaches, may enforce the food industry to decrease the sodium content in food products. 

## Figures and Tables

**Table 1:** Sociodemographic and physical characteristics of the participants (n = 467).

Characteristics	*n*	(%)
**Age (years)**		
20–29	294	63.0
30–39	91	19.5
≥40	82	17.5
**Sex**		
Male	186	39.8
Female	281	60.2
**Marital status**		
Single	196	42.0
Married	271	58.0
**Education level**		
High-school/Diploma	229	49.0
University degree/Postgraduate degree	238	51.0
**Family income (SR)**		
<5000	243	52.0
5000–10,000	102	21.9
>10,000	122	26.1
**City of residence**		
Madinah	368	78.8
Jeddah	99	21.2
**Weight status (BMI in kg/m^2^)**		
Underweight (<18.5)	40	8.57
Healthy weight (18.5–24.99)	179	38.3
Overweight (25–29.99)	122	26.1
Obese (≥30)	126	27.0
**Blood pressure (mm Hg)**		
Normal (<120/80)	158	33.8
Elevated (systolic 120–129 and diastolic <80)	42	8.99
Stage 1 hypertension (systolic 130–139 or diastolic 80–89)	186	39.8
Stage 2 hypertension (systolic >140 or diastolic ≥90)	81	17.3

SR: Saudi Riyal (SR 3.75 = $1.00); BMI: body mass index.

**Table 2:** Salt-related knowledge and practices stratified by sex (n = 467).

	Variable	Males (*n* = 186)	Females (*n* = 281)	Total (*n* = 467)	*p*
**Salt-related knowledge**					
1	Could high salt intake in the diet adversely affect health?				
	Yes ^a^	169 (90.9)	274 (97.5)	443 (94.9)	0.002 *
	No	17 (9.10)	7 (2.50)	24 (5.10)	
Could high salt intake increase the risk for……					
2	Hypertension?				
	Yes ^a^	163 (87.6)	262 (93.2)	425 (91.0)	0.038 *
	No	23 (12.4)	19 (6.80)	42 (9.00)	
3	Osteoporosis?				
	Yes ^a^	23 (12.4)	35 (12.5)	58 (12.4)	0.977
	No	163 (87.6)	246 (87.5)	409 (87.6)	
4	Stomach cancer?				
	Yes ^a^	9 (4.80)	15 (5.30)	24 (5.10)	0.811
	No	117 (95.2)	266 (94.7)	443 (94.9)	
5	Kidney stones?				
	Yes ^a^	70 (37.6)	155 (55.2)	225 (48.2)	<0.001 *
	No	116 (62.4)	126 (44.8)	242 (51.8)	
**Salt-related practices**					
1	Sodium intake within the recommendation of the American Heart Association (<2,300 mg/day)	15 (8.10)	29 (10.3)	44 (9.40)	0.414
	Sodium intake above the recommendation of the American Heart Association (≥2,300 mg/day)	171 (91.9)	252 (89.7)	423 (90.6)	
2	**Addition of salt to food during cooking**				
	Always/Usually/Sometime	177 (95.2)	270 (96.1)	447 (95.7)	0.629
	Rarely/Never	9 (4.80)	11 (3.90)	20 (4.30)	
3	**Addition salt to food at the table**				
	Always/Usually/Sometime	46 (24.7)	49 (17.5)	95 (20.4)	0.058
	Rarely/Never	140 (75.3)	231 (82.5)	371 (79.6)	
4	**Previously tried reducing salt intake**				
	Yes	75 (40.3)	134 (47.7)	209 (44.8)	0.117
	No	111 (59.7)	147 (52.3)	258 (55.2)	
5	**Previously tried using salt alternatives**				
	Yes	2 (1.10)	12 (4.30)	14 (3.00)	0.047 *
	No	184 (98.9)	269 (95.7)	453 (97.0)	
6	**Fast food consumption**				
	Once a week or less	41 (22.0)	131 (46.6)	172 (36.8)	<0.001 *
	Twice a week or more	145 (78.0)	150 (53.4)	295 (63.2)	

All values presented are frequencies (%); ^a^ Correct response; * Values of *p* < 0.05 were considered significant across males and females.

**Table 3:** Association between salt-related knowledge and practices and sociodemographic and physical characteristics (*n* = 467).

Characteristics	Knowledge Score Out of 5 Mean (SD)	Practice Score Out of 6 Mean (SD)
**Age (years)**		
20–29	3.29 (0.79)	1.60 (0.96)
30–39	3.16 (0.73)	1.73 (0.99)
≥40	3.32 (0.66)	2.37 (0.94)
*p*-value	0.211	<0.001 *
**Sex**		
Male	3.09 (0.83)	1.50 (0.94)
Female	3.39 (0.68)	1.93 (1.00)
*p*-value	<0.001 *	<0.001 *
**Marital status**		
Single	3.25 (0.74)	1.97 (1.00)
Married	3.28 (0.78)	1.60 (0.97)
*p*-value	0.653	<0.001 *
**Education level**		
High-school/Diploma	3.24 (0.77)	1.74 (0.96)
University degree/Postgraduate degree	3.29 (0.75)	1.78 (1.04)
*p*-value	0.536	0.684
**Family income (SR)**		
<5000	3.29 (0.76)	1.69 (1.01)
5000–10,000	3.14 (0.81)	1.80 (0.96)
>10,000	3.33 (0.72)	1.85 (1.01)
*p*-value	0.205	0.313
**City of residence**		
Madinah	3.24 (0.73)	1.80 (0.97)
Jeddah	3.35 (0.82)	1.57 (1.10)
*p*-value	0.178	0.043 *
**Weight status (BMI in kg/m^2^)**		
Underweight (<18.5)	3.20 (0.72)	1.74 (0.82)
Healthy weight (18.5–4.99)	3.32 (0.77)	1.59 (1.03)
Overweight (25–29.99)	3.16 (0.80)	1.71 (0.99)
Obese (>30)	3.31 (0.72)	2.04 (0.96)
*p*-value	0.268	0.001 *
Blood pressure (mm Hg) *		
Normal (<120/80)	3.32 (0.76)	1.82 (0.98)
Elevated (systolic 120–129 and diastolic <80)	3.17 (0.76)	1.48 (1.05)
Stage 1 hypertension (systolic 130–139 or diastolic 80–89)	3.31 (0.72)	1.73 (1.03)
Stage 2 hypertension (systolic >140 or diastolic ≥90)	3.12 (0.84)	1.85 (0.92)
*p*-value	0.213	0.139

SD: standard deviation; SR: Saudi Riyal (SR 3.75 = $1.00); * Values of *p* < 0.05 were considered significant.

**Table 4:** Multiple linear regression analysis of salt-related knowledge and practice scores in relation to sodium intake, blood pressure, and body mass index (BMI).

	Beta Estimate	Standard Error	*p*	95% CI	F	R-Square
**Sodium intake (mg/day)**						
Salt-related knowledge	−88.3	163	0.588	−408 to 232	2.38	0.01
Salt-related practices	−457	123	<0.001 *	−699 to −215	4.65	0.05
**Systolic blood pressure (mm Hg)**						
Salt-related knowledge	−0.33	0.70	0.643	−1.71 to 1.06	35.1	0.13
Salt-related practices	0.39	0.55	0.480	−0.69 to 1.46	14.9	0.14
**Diastolic blood pressure (mm Hg)**						
Salt-related knowledge	−0.37	0.58	0.522	−1.51 to 0.77	4.13	0.02
Salt-related practices	0.35	0.45	0.444	−0.54 to 1.23	1.80	0.02
**BMI (BMI in kg/m^2^)**						
Salt-related knowledge	0.60	0.36	0.119	−0.15 to 1.26	9.74	0.04
Salt-related practices	0.56	0.25	0.026 *	0.07 to 1.05	28.2	0.23

Models of salt-related knowledge were adjusted for sex; Models of practices related to salt were adjusted for age, sex, marital status, and city of residence; * Values of *p* < 0.05 were considered significant.
